# Hepatitis mortality in Brazil and regions, 2001–2020: temporal trend and spatial analysis

**DOI:** 10.1590/1980-549720230029

**Published:** 2023-07-03

**Authors:** Laryssa Fialho de Oliveira Sousa, Evelen Rouse de Souza Santos, Rayssa Mendonça Oliveira, Renata Lima Batalha Andrade, Jefferson Felipe Calazans Batista, Sonia Oliveira Lima

**Affiliations:** I Universidade Tiradentes – Aracaju (SE), Brazil.

**Keywords:** Time series studies, Spatial analysis, Hepatitis, chronic, Hepatitis, viral, human, Estudos de séries temporais, Análise espacial, Hepatite crônica, Hepatite viral humana

## Abstract

**Objective:**

To analyze the spatial distribution and the temporal trend of the hepatitis mortality rate in Brazil from 2001 to 2020.

**Methods:**

Ecological, temporal, and spatial study on mortality from hepatitis in Brazil with data from the Mortality Information System (*Sistema de Informações sobre Mortalidade* – SIM/DATASUS). Information was stratified by year of diagnosis, region of the country, municipalities (of residence). Standardized mortality rates (SMR) were calculated. The temporal trend was estimated by Prais-Winsten regression and the spatial distribution by the Global Moran Index (GMI).

**Results:**

The highest SMR means in Brazil were for Chronic viral hepatitis with 0.88 deaths per 100,000 inhabitants (SD=0.16), followed by Other viral hepatitis with 0.22/100,000 (SD=0.11). In Brazil, the temporal trend of mortality from Hepatitis A was −8.11% per year (95%CI −9.38; −6.82), while for Hepatitis B it was −4.13% (95%CI −6.03; −2.20), of Other viral hepatitis of −7.84% (95%CI −14.11; −1.11) and of Unspecified Hepatitis −5.67% per year (95%CI −6.22; −5.10). Mortality due to chronic viral hepatitis increased by 5.74% (95%CI 3.47; 8.06) in the North and 4.95% in the Northeast (95%CI 0.27; 9.85). The Moran Index (I) for Hepatitis A was 0.470 (p<0.001), for Hepatitis B 0.846 (p<0.001), Chronic viral hepatitis=0.666 (p<0.001), other viral hepatitis=0.713 (p<0.001), and Unspecified Hepatitis=0.712 (p<0.001).

**Conclusion:**

The temporal trend of hepatitis A, B, other viral, and unspecified hepatitis was decreasing in Brazil, while mortality from chronic hepatitis was increasing in the North and Northeast.

## RESUMO

**Objetivo:**

Analisar a distribuição espacial e a tendência temporal da taxa de mortalidade por hepatites no Brasil no período de 2001 a 2020.

**Métodos:**

Estudo ecológico, temporal e espacial sobre a mortalidade por hepatites no Brasil com dados do Sistema de Informações sobre Mortalidade (SIM/Datasus). As informações foram estratificadas por ano do diagnóstico, região do país, municípios (de residência). Foram calculadas as taxas padronizadas de mortalidade (TPM). A tendência temporal foi estimada pela regressão de Prais-Winsten e a distribuição espacial pelo Índice Global de Moran (IGM).

**Resultados:**

As maiores médias da TPM no Brasil foram para hepatite viral crônica, com 0,88 mortes para cada 100 mil habitantes (desvio padrão — DP=0,16), seguida de outras hepatites virais, com 0,22/100 mil (DP=0,11). No Brasil, a tendência temporal da mortalidade por hepatite A foi de −8,11% ao ano (intervalo de confiança de 95% — IC95% −9,38; −6,82), enquanto por hepatite B foi de −4,13% (IC95% −6,03; −2,20); de outras hepatites virais, foi de −7,84% (IC95% −14,11; −1,11) e de hepatite não especificada, de −5,67% ao ano (IC95% −6,22; −5,10). A mortalidade por hepatite viral crônica cresceu 5,74% (IC95%3,47; 8,06) no norte e 4,95% no nordeste (IC95% 0,27; 9,85). O Índice de Moran (I) para hepatite A foi de 0,470 (p<0,001), para hepatite B de 0,846 (p<0,001), hepatite viral crônica=0,666 (p<0,001), outras hepatites virais=0,713 (p<0,001) e hepatites não especificadas=0,712 (p<0,001).

**Conclusão:**

A tendência temporal das hepatites A, B, de outras hepatites virais e das não especificadas foi de diminuição no Brasil, enquanto a mortalidade por hepatites crônicas foi de crescimento nas Regiões Norte e Nordeste.

## INTRODUCTION

Hepatitis is defined as inflammation in the liver, which can onset from different etiologies, acutely or chronically. One of the main etiologies is viral, in which hepatotropic viruses such as hepatitis A, B, C, D, and E stand out. Out of those ones, hepatitis caused by the B, C, and delta tend to become more chronic and may progress to liver cirrhosis^1^, representing important causes of liver transplantation in industrialized countries^2^.

With regard to the mortality rate of viral hepatitis, data from the World Health Organization (WHO) reveal a higher rate in the Western Pacific, Southeast Asia, and African regions, respectively, with the lowest rate in the American region^3^.

In 2015, also according to the WHO, 257 million people lived with chronic hepatitis B, and 71 million with chronic hepatitis C, which accounted for 96% of the 1.34 million deaths that year^3^. In this context, the prevalence of the hepatitis C virus (HCV) varied around the world, with 0.2 to 3% of cases in Europe and the United States, reaching 5% in the African continent^2^. With regard to the incidence of hepatitis B virus (HBV), its decrease since the implementation of the vaccine stands out, which can be observed in the United States between the years 2008 to 2012, when there was a 28% reduction in the number of cases, with an annual rate of 0.9 cases per 100,000 inhabitants^4^.

According to the Ministry of Health, viral hepatitis is highly transmissible, with an estimated 240 million chronic infections worldwide for HBV. This etiology is globally responsible for approximately 780,000 deaths per year^5^. In Brazil, it is estimated that hepatitis A has a higher concentration in the Northeast and North regions, which together account for 55.4% of total cases, while hepatitis B is more prevalent in the Southeast and South regions, with 66% of cases. Hepatitis C is predominant in the Southeast, with 58.9%, and Hepatitis D in the North, with 74.8% of cases^6^.

In this scenario, the National Viral Hepatitis Program (*Programa Nacional de Hepatites Virais* – PNHV) was implemented in Brazil in 2002, with the aim of creating guidelines and strategies for preventing and combating hepatitis. In addition to being able to know the epidemiological reality of these infections through the projects Sentry of Pregnant Women (*Sentinela de Gestantes*), Sentry of the Armed Forces (*Sentinela das Forças Armadas*), and Household Survey of Viral Hepatitis (*Inquérito Domiciliar de Hepatites Virais*)^7^, hepatitis is responsible for a significant number of cases in Brazil, with different epidemiological behavior in the major regions. Current public policies encourage preventive care, measures that should promote the reduction of deaths from this condition^6^. However, as there are few studies that assess mortality from hepatitis at the national level, the objective was to evaluate the temporal trend and spatial dependence of mortality from hepatitis in Brazil and its regions in the period from 2001 to 2020.

## METHODS

Ecological time series study, with a quantitative approach, of a descriptive and exploratory nature, which used data referring to mortality from hepatitis in Brazil and its regions in the period between 2001 and 2020. The codes of the International Classification of Diseases and Conditions (ICD-10) relating to hepatitis were:

- B15 — acute hepatitis A
- B16 — acute hepatitis B
- B17 — other acute viral hepatitis
- B18 — chronic viral hepatitis
- B19 — unspecified viral hepatites (US)

The information was taken from the Brazilian Mortality Information System (*Sistema de Informações sobre Mortalidade* – SIM) of the Department of Informatics of the Unified Health System (*Departamento de Informática do Sistema Único de Saúde* – Datasus/TABNET). They were stratified based on death by residence, region, municipalities, year of death (2001–2020) and category of the 10^th^ Revision of the International Classification of Diseases — ICD-10 (B15 to B19). Blank and ignored data were removed from the survey.

The standardization of the mortality rate followed the Curtin and Klein methods of the National Center for Health Statistics (NCHS)^8^. Standardization was calculated by age groups every ten years, stratified by ICD-10 category in each region of the country. The direct method was adopted, comprising the standard population to the world according to WHO (2000–2025)^9^.

The population bases used resulted from the 2000 and 2010 population censuses, as well as the census projections between 2001 and 2020, by the Brazilian Institute of Geography and Statistics (*Instituto Brasileiro de Geografia e Estatística* – IBGE)^10^.

The raw values of the occurrences were arranged by means of absolute and relative frequency, in order to compare the standardized mortality rates (SMR) between regions and hepatitis when calculating the annual mean and standard deviation (SD) of the coefficients.

The temporal trend analysis of the present study followed the standards established in the literature^11^. For the calculation, the linear regression model with Prais-Winsten autocorrelation correction was used. The base estimation formula is


$$Y=b_{0}+b_{1}X$$

where the value of b_0_ corresponds to the intersection of the vertical axis and the line; b_1_ is the slope of the line; Y values are the same as those of the time series — in the case of this study, SMR; and X is the time scale (year). For every change in X, the value of Y changes in b_1_; however, as the values between the variables are measured on different scales (time and rate), a percentage estimator is calculated^11^.

This measure uses TPM values transformed into base 10 logarithmic values. According to Antunes and Cardoso^11^, this makes it possible to reduce the heterogeneity of the variance of the residues and correct deviations from normality. To estimate the annual percentage change (APC) and its respective 95% confidence interval (95%CI), the following formulas were used:


$$\begin{array}{l} APC=\left[-1+10^{b1}\right]\times 100\%\\ CI_{minimum}95\%=\left[-1+10^{CI\;min.\;b1}\right]\times 100\%\\ CI_{maximum}95\%=\left[-1+10^{CI\;max.\;b1}\right]\times 100\% \end{array}$$

These indicators are used to describe and quantify the trend. Negative results indicate a decrease, positive results indicate an increase (p<0.05), if there is statistical significance (p<0.05), and the absence of significance (p>0.05) corresponds to a stationary trend^11^. After correcting for autocorrelation, Durbin-Watson values between 1.5 and 2.5 were considered satisfactory.

Spatial analysis was performed using Global and Local Moran's Index (GMI/LMI). For the estimate, the units of analysis were the municipalities in Brazil that had deaths from hepatitis. The degree of proximity was determined by the weight matrix, using the queen contiguity criterion. This weight matrix identifies the first order neighbors of a municipality in a 360° range^12–14^. GMI calculates the spatial dependence across the country, while the LMI allows identifying the correlation for each municipality. Thus, a Univariate Local Moran was performed using Spatial Empirical Bayesian Smoothing (SEBS). To estimate the significance of the indices, the pseudo significance test was used through 9,999 permutations^13^. The cartographic demonstration was performed using the Local Indicators of Spatial Association (LISA) map, which categorizes municipalities through Local Moran's I.

The cartographic base (territorial grids) was provided by IBGE in the 2020 version. The projection corresponded to the Universal Transverse Mercator (UTM) system, using the Geodetic Reference System for the Americas 2000 model (SIRGAS 2000).

The programs used were Stata 17 for trend calculations, Microsoft Excel 2019 for descriptive analysis and calculations of rate-type measurements, and GeoDa version 1.20 for the Moran Index. Significance and spatial levels of 5% (p<0.05) and 1% (p<0.01) were adopted for the trend model, in order to reduce the problem of multiple comparisons.

The present study waived the appreciation of the Ethics and Research Committee for presenting, as a source of information, secondary data open to the public.

## RESULTS

In Brazil, in the last 20 years, a total of 49,831 deaths from hepatitis were recorded. In this scenario, the region with the highest mortality was the Southeast, with 50.95% of deaths (n=25,389). On the other hand, the region with the lowest mortality was the Central-West, with 5.36% of deaths (n=2,669). The Northeast Region ranked third, with 13.42% of deaths (n=6,685).

With regard to SMR, the highest Brazilian means were for chronic viral hepatitis, with 0.88 deaths per 100,000 inhabitants (SD=0.16), followed by other viral hepatitis with 0.22/100 thousand (SD=0.11), hepatitis B with 0.14/100 thousand (SD=0.04), unspecified hepatitis with 0.12/100 thousand (SD=0.04), and hepatitis A with a mean of 0.02/100 thousand (SD=0.01). In chronic viral hepatitis, the South and Southeast Regions exceeded the national mean, with 1.38 (SD=0.25) and 1.06 (SD=0.23) per 100 thousand inhabitants. The North Region exceeded the Brazilian mean for hepatitis A (SMR=0.06; SD=0.04), hepatitis B (SMR=0.45; SD=0.10), other hepatitis (SMR=0.41; SD=0.18), and unspecified hepatitis (SMR=0.23; SD=0.13). The Supplementary Material Tables present SMR by year, region, and ICD-10 cause.

**Figure 1:**
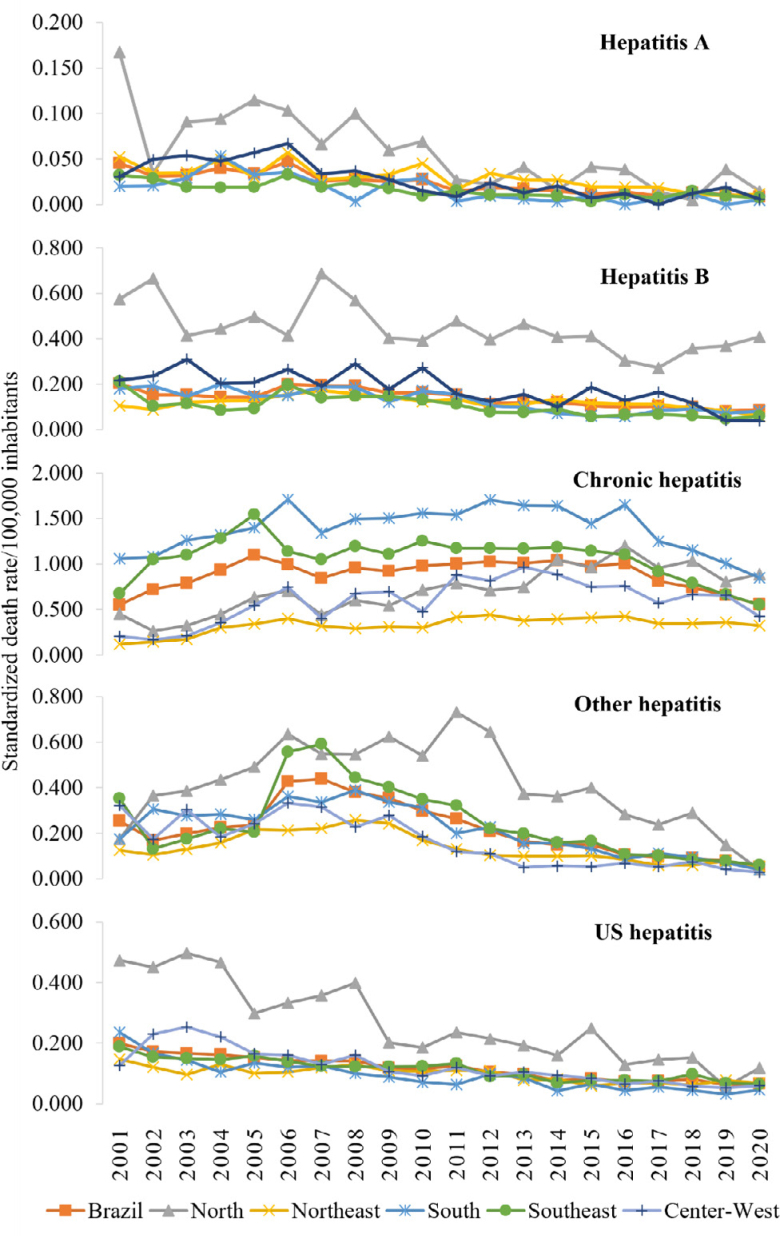
Standardized mortality rate (per 100,000 inhabitants) due to hepatitis in Brazil and its regions, from 2001 to 2020.

The temporal trend in Brazil decreased for all types of hepatitis, except chronic viral, which was stationary. In the North Region, there was an increase in SMR for chronic viral hepatitis and a decrease for hepatitis A, B, and US. In the Northeast, the pattern decreased for hepatitis A and US and increased for chronic hepatitis. The Southeast showed a decrease in all causes, except for chronic hepatitis, which was stationary, while there is a decrease in hepatitis B in the South and Center-West, other hepatitis and US (Table 1, Figure 1).

**Table 1:** Time trend of the standardized mortality rate due to hepatitis in Brazil and its regions, 2001–2020.

Location/type		APR (%)	(95%)CI		p-value	Corrected D-W	Interpretation
			Lower	Upper			
Brazil							
	Hepatitis A	-8.11	-9.38	-6.82	<0.001	1.983	Decreased
	Hepatitis B	-4.13	-6.03	-2.20	<0.001	1.839	Decreased
	Other viral hepatitis	-7.84	-14.11	-1.11	0.026	1.359	Decreased
	Chronic viral hepatitis	-0.003	-4.45	4.65	0.999	1.448	Stationary
	Unspecified viral hepatitis	-5.67	-6.22	-5.10	<0.001	1.976	Decreased
North							
	Hepatitis A	-10.70	-14.34	-6.92	<0.001	2.049	Decreased
	Hepatitis B	-2.58	-4.07	-1.07	0.002	1.902	Decreased
	Other viral hepatitis	-6.99	-18.88	6.65	0.281	1.238	Stationary
	Chronic viral hepatitis	5.74	3.47	8.06	<0.001	1.872	Increased
	Unspecified viral hepatitis	-8.40	-9.84	-6.94	<0.001	2.151	Decreased
Northeast							
	Hepatitis A	-6.88	-8.74	-4.98	<0.001	1.933	Decreased
	Hepatitis B	-2.59	-6.34	1.30	0.176	1.536	Stationary
	Other viral hepatitis	-4.64	-9.89	0.91	0.094	1.270	Stationary
	Chronic viral hepatitis	4.95	0.27	9.85	0.039	1.462	Increased
	Unspecified viral hepatitis	-3.82	-5.33	-2.28	<0.001	2.051	Decreased
Southeast							
	Hepatitis A	-7.30	-10.19	-4.31	<0.001	1.989	Decreased
	Hepatitis B	-5.48	-8.21	-2.67	0.001	1.954	Decreased
	Other viral hepatitis	-8.10	-15.12	-0.49	0.039	1.797	Decreased
	Chronic viral hepatitis	-1.35	-5.24	2.71	0.488	1.676	Stationary
	Unspecified viral hepatitis	-5.02	-6.03	-4.01	<0.001	1.983	Decreased
South							
	Hepatitis A	0.80	-9.87	12.73	0.883	2.075	Stationary
	Hepatitis B	-5.46	-7.93	-2.92	<0.001	1.951	Decreased
	Other viral hepatitis	-7.86	-13.22	-2.18	0.010	1.928	Decreased
	Chronic viral hepatitis	-0.94	-4.67	2.94	0.612	2.061	Stationary
	Unspecified viral hepatitis	-7.96	-9.12	-6.79	<0.001	1.998	Decreased
Center-West							
	Hepatitis A	-5.55	-12.99	2.53	0.161	1.960	Stationary
	Hepatitis B	-7.26	-10.77	-3.61	0.001	1.851	Decreased
	Other viral hepatitis	-11.50	-15.45	-7.36	<0.001	2.081	Decreased
	Chronic viral hepatitis	4.88	-1.23	11.36	0.113	1.871	Stationary
	Unspecified viral hepatitis	-6.99	-8.37	-5.58	<0.001	1.557	Decreased

APR: annual percentagem rate; CI: confidence interval; D-W: Durbin-Watson.

**Figure 2:**
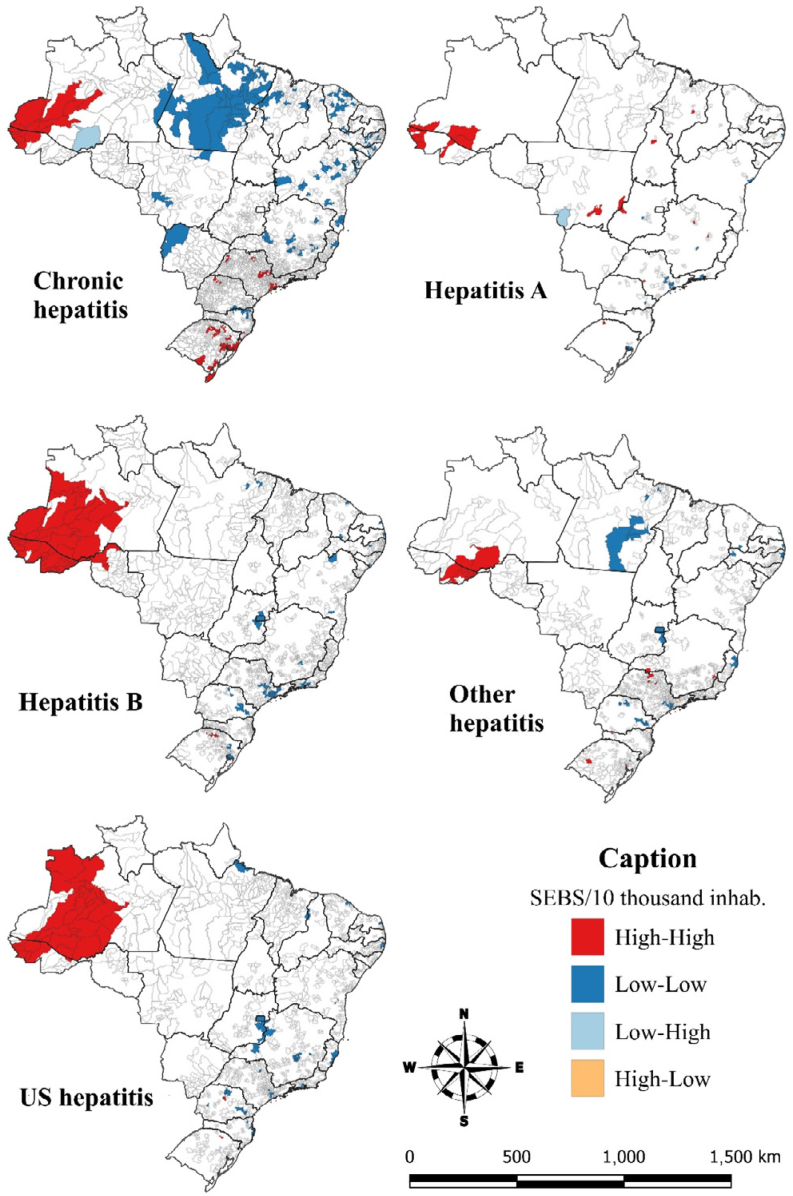
Local Indicators of Spatial Association (LISA) map of Spatial Empirical Bayesian Hepatitis Mortality Rates, according to the 10^th^ Revision of the International Classification of Diseases in Brazil and its regions, from 2001 to 2020.

Global Moran's analysis pointed to direct spatial dependence in all hepatitis. The Moran Index (I) for hepatitis A was 0.470 (p<0.001), for hepatitis B 0.846 (p<0.001), for chronic viral hepatitis 0.666 (p<0.001), for other viral hepatitis 0.713 (p <0.001), and for unspecified hepatitis 0.712 (p<0.001). In all estimates, the North Region, specifically Acre and Amazonas, presented clusters with high SEBS, while clusters with low rates of chronic hepatitis were identified in the state of Pará (Figure 2).

## DISCUSSION

Among hepatitis, chronic viral led with the highest death rates in all regions of the country. This can be justified by the fact that chronic infections of hepatitis B or C are important predictors of the development of cirrhosis and hepatocellular carcinoma^15^. Cirrhosis is the main factor for the development of carcinoma and this, in turn, is the most common primary liver cancer, accounting for 90% of cases worldwide^16^.

Therefore, public campaigns that encourage vaccination, the main form of prevention against hepatitis B, are essential. This importance is proven when one finds that, after a decade of the implementation of this vaccine for adults in the United States, there was an 82% reduction in incidence of acute hepatitis B, and this country is now considered at low risk for infection by this virus^4^. In addition, the efficacy of the vaccine for the prevention of hepatitis B is also very well documented in Taiwan, a Republic where the number of HBsAg-positive individuals younger than 20 years decreased from 9.8% in 1984 to 0.6% in 2004, resulting in a significant drop in infection-related complications. In the same sense, the lack of adequate and comprehensive vaccination for adults resulted in an increase in the incidence of HBV infection in sub-Saharan Africa. This region has high rates of the disease in adults, a fact explained by the exclusion of those born before 1995, the year in which the immunization program was implemented for those born after that year^17^.

Brazil showed a decreasing trend in mortality in almost all types of hepatitis. This fact can be explained by the universalization of the vaccine against hepatitis B, by the higher quality in the hemotherapy processes, by the creation, in 2002, of the PNHV and by a subsequent offer of more effective tests in the detection of the C virus^18^, in addition to the offer of new treatments^5^. Hepatitis B vaccine became universal for children under one year of age in 1998 and for people under 20 years of age in 2001. However, even with vaccination coverage below the recommended level, the complete three-dose regimen in children increased globally from 3% in 1992 to 85% in 2019, which contributed to the drop in the number of deaths^19^. Added to this fact, it is also worth mentioning the determination of Ordinance 1.376/93, reinforced by Resolution 343/2001 of the Ministry of Health (MoH), on the performance of serological screening tests in hemotherapy services for hepatitis B and other diseases, through technical norms for the collection, processing, and transfusion of blood, which significantly reduced the number of people infected by the hepatitis B and C viruses via transfusion^20^.

With regard to PNHV, some objectives stand out, including the need to know the epidemiological reality of hepatitis in Brazil, since these infections were not subject to compulsory notification until 1996, the year in which they began to make up the list. In addition to this objective, it is worth mentioning the lack at the time of carrying out a diagnosis of the situation of the laboratory and outpatient care network, as well as sensitizing state and municipal managers and communicators about the situation of viral hepatitis, with the aim of elaborating and always keeping up to date the Brazilian Consensus for the Treatment of Viral Hepatitis^7^.

In 2014, the Ministry of Health, as recommended by the World Health Organization, began to provide access to direct-acting antivirals without interferon for the treatment of hepatitis C. Treatment was offered to approximately 70,000 people from 2014 to 2017^18^. There was also the publication, in 2017, of the new Clinical Protocols and Therapeutic Guidelines (*Protocolos Clínicos e Diretrizes Terapêuticas* – PCDT) for hepatitis C and coinfections, which now cover new drugs and expand the inclusion of special groups^5^. These facts may explain the reduction in deaths from viral hepatitis in Brazil.

Hepatitis A mortality showed a downward trend in the country and in the North, Northeast, and Southeast regions. This pattern may reflect two major changes:

1. Change in the standard of living of the population, represented by greater economic power and access to basic sanitation, which reduces incidence and consequent mortality^21^;
2. Insertion of the vaccination schedule for hepatitis A in the National Immunization Program since 2014^22^.

The Southeast was the only region to show a decreasing trend for almost all types of hepatitis, except for chronic viral hepatitis, which was stationary. The downward trend in deaths in this region can be explained by the higher detection of viral hepatitis, possibly due to a better-quality epidemiological surveillance system^23^. This greater detection is achieved by the greater number of reference laboratories for hepatitis tests in the Southeast^7^. This reduction is also explained by the high number of people vaccinated in the region, which, together with the south, had the highest vaccination coverage against the hepatitis B virus between 2017 and 2018, according to data from Datasus^24^. This demonstrates that, following vaccination protocols, the incidence of this condition can be reduced and, when it is present, it is possible, with adequate treatment, to reduce mortality due to viral hepatitis.

The temporal trend of mortality from chronic viral hepatitis was stationary in most regions, except for the North and Northeast, where there was growth. It is known that chronic hepatitis can persist for years or even decades and, despite causing mild liver damage, its constancy deteriorates the liver and can cause other problems such as cirrhosis, liver failure, cancer, among others. Reduced access to diagnostic and treatment services for these conditions in the North and Northeast regions may explain the growth trend^25,26^. Therefore, prevention (hepatitis C) and vaccination (hepatitis B) policies must be intensified, especially in priority regions.

The states of Amazonas and Acre presented spatial conglomerates with high mortality rates in all hepatitis. This spatial arrangement can be explained in the North Region by the high prevalence of seropositivity for the hepatitis Delta virus (HDV), which reached 77% of national cases. HDV infection only occurs in humans already infected with HBV, often leading to the rapid development of serious liver conditions such as cirrhosis, hepatocellular cancer, and fulminant hepatitis. Despite these high endemic indicators in the north of the country, this condition is still neglected by health services^27^.

It was evident that, in the 2001-2020period, mortality rates due to hepatitis decreased, a fact that can be attributed mainly to prevention policies, such as vaccination. However, the chronicity of hepatitis still draws attention, given its high mortality rates and its growth pattern in some regions. The lack of information and interest, the difficulty in scheduling periodic appointments, the indifference of some health professionals, the shortage of doctors, and the low supply of services are factors that reduce the possibility of effective treatment for an easily diagnosed disease^28^. It is imperative to recognize Brazil's health, social, economic, demographic, and political vulnerabilities, and thereby improve public policies aimed at preventing and treating these diseases through constant education of the population, improvement of health professionals and of the provision of services to ultimately reduce mortality rates from viral hepatitis.

The present study has some limitations, such as the underreporting of deaths from hepatitis, especially chronic viral infections (HBV and HCV), due to progression to carcinoma, with subsequent coding as “liver cancer”, as well as the non-diagnosis and coding of the possible underlying etiologies^29^. Another limiting factor is the autocorrelation of residuals. The Prais-Winsten technique did not satisfactorily correct the autocorrelation of some estimates. This, depending on its value, can lead to misinterpretation both for more (overestimation) and for less (underestimation), when, in fact, the estimate would be stationary. This must be borne in mind when considering these results. However, its importance is emphasized for the epidemiological mapping of mortality from viral hepatitis, pointing out the regions that need to improve existing public policies.

In the studied period, considerable standardized mortality rates due to hepatitis were observed in Brazil, with chronic viral infections responsible for most of these deaths. The temporal trend of hepatitis A, B, other viral hepatitis, and US was decreasing in Brazil, while the trend of mortality due to chronic hepatitis was increasing in the north and northeast. Spatial clusters with high mortality rates were identified in the Amazon and Acre regions, in all hepatitis cases.
